# *In vivo* comparison of the charge densities required to evoke motor responses using novel annular penetrating microelectrodes

**DOI:** 10.3389/fneng.2015.00005

**Published:** 2015-05-12

**Authors:** Emma K. Brunton, Bjorn Winther-Jensen, Chun Wang, Edwin B. Yan, Saman Hagh Gooie, Arthur J. Lowery, Ramesh Rajan

**Affiliations:** ^1^Monash Vision Group, Monash UniversityClayton, VIC, Australia; ^2^Department of Electrical and Computer Systems Engineering, Monash UniversityClayton, VIC, Australia; ^3^Department of Physiology, Monash UniversityClayton, VIC, Australia; ^4^Department of Materials Engineering, Monash UniversityClayton, VIC, Australia

**Keywords:** neural prosthesis, microelectrodes, charge injection capacity, *in vivo*, neural stimulation

## Abstract

Electrodes for cortical stimulation need to deliver current to neural tissue effectively and safely. We have developed electrodes with a novel annular geometry for use in cortical visual prostheses. Here, we explore a critical question on the ideal annulus height to ensure electrical stimulation will be safe and effective. We implanted single electrodes into the motor cortex of anesthetized rats and measured the current required to evoke a motor response to stimulation, and the charge injection capacity (CIC) of the electrodes. We compared platinum iridium (PtIr) electrodes with different annulus heights, with and without a coating of porous titanium nitride (TiN). Threshold charge densities to evoke a motor response ranged from 12 to 36 μC.cm^-2^.ph^-1^. Electrodes with larger geometric surface areas (GSAs) required higher currents to evoke responses, but lower charge densities. The addition of a porous TiN coating did not significantly influence the current required to evoke a motor response. The CIC of both electrode types was significantly reduced *in vivo* compared with *in vitro* measurements. The measured CIC was 72 and 18 μC.cm^-2^.ph^-1^ for electrodes with and without a TiN coating, respectively. These results support the use of PtIr annular electrodes with annulus heights greater than 100 μm (GSA of 38, 000 μm^2^). However, if the electrodes are coated with porous TiN the annulus height can be reduced to 40 μm (GSA of 16,000 μm^2^).

## Introduction

Stimulation of nerve cells for the restoration of brain and sensory deficits has been successfully implemented, most prominently with the cochlear implant (Tyler et al., 1988; Svirsky et al., 2000; Cogan, 2008) and in deep brain stimulation (Breit et al., 2004). This has been extended to vision restoration for the blind by the development of prostheses that stimulate nerve cells at some point along the visual pathway – the retina (Schwahn et al., 2001; Zrenner et al., 2009; Humayun et al., 2012; Shepherd et al., 2013), the optic nerve (Delbeke et al., 2002; Veraart et al., 2003), and the visual cortex (Dobelle, 1999; Normann et al., 1999; Troyk et al., 2005; Lowery, 2013) – and produce the perception of spots of light, ‘phosphenes’ (Weiland and Humayun, 2008; Shepherd et al., 2013). Phosphenes can then be used to provide useful “visual” information to the recipient (Delbeke et al., 2002). A cortical prosthesis has the potential to treat all types of late blindness except when caused by direct damage to the brain (Lewis et al., 2015) and hence the Monash Vision Group is developing a cortical visual prosthesis with electrodes that penetrate the cortical surface to target layer 4 of the primary visual cortex (Lowery, 2013; Lewis et al., 2015).

Regardless of the implant’s location, the electrodes must be capable of stimulating safely and effectively. Platinum (Pt) electrodes have a proven track record for use in stimulating prostheses in human tissue, and have been safely used in cochlear prostheses for decades. However, for a penetrating cortical prosthesis the material also needs to be strong enough to penetrate the cortical tissue. Pure Pt is too soft to do so (Brummer et al., 1983) and hence the electrode shafts need to be made from stronger materials, e.g., platinum iridium (PtIr) alloys, to ensure that electrodes do not bend during insertion (Brummer et al., 1983).

The charge injection capacity (CIC) for Pt and PtIr alloy electrodes has been estimated to be 50–150 μC.cm^-2^.ph^-1^ (Rose and Robblee, 1990). Cochlear implant electrodes have large surface areas of approximately 0.4 mm^2^ and the charge injections used in clinical stimulation normally do not exceed 20 μC.cm^-2^.ph^-1^ (Cogan, 2008). However, these charge densities are unlikely to be appropriate for a visual cortical prosthesis with small penetrating microelectrodes; Schmidt et al. (1996) showed that the threshold for the human perception of phosphenes when using small surface area (200 μm^2^) penetrating microelectrodes to stimulate visual cortex is about 190–2500 μC.cm^-2^.ph^-1^, a range much greater than the aforementioned safe charge density per phase for platinum electrodes. Electrodes with larger surface areas require higher currents but lower charge densities to deliver effective stimulation (Brummer et al., 1983). Thus if PtIr alloy electrodes are to be used in a cortical visual prosthesis, modifications must be made to the electrode geometry to increase the electrode’s surface area to ensure effective charge densities are below the CIC of PtIr (Brummer et al., 1983). Alternatively coatings capable of improving the CIC of the electrodes can be applied to the electrodes.

Previously we have reported on the use of novel annular electrodes in a chronic cortical implant (Wang et al., 2013) instead of the more commonly used ‘tip’ electrode (e.g., Utah electrode array) or a multitrode electrode with multiple disk shaped contacts embedded along the shaft (Michigan electrode array). Modeling studies have shown that pointed tip electrodes have a large peak in current density at the very tip of the electrode (McIntyre and Grill, 2001; Brunton et al., 2012), while annular electrodes still show current density peaks at the electrode-insulation boundary; in contrast, the peak current density is greatly reduced on the annular electrode (Brunton et al., 2012). Both the tip electrodes and disk electrodes on the multitrode have relatively small surface areas of less than 5000 μm^2^ (Davis et al., 2012) and consequently require large charge densities to evoke behavioral responses. These large charge densities can result in tissue and electrode damage (McCreery et al., 1990; Shannon, 1992). In comparison the annular electrodes have a surface area greater than 7000 μm^2^ and require much lower charge densities to evoke stimulation associated responses (Wang et al., 2013). However, as the surface area of these electrodes is larger, the stimulation will be less focused. In order to improve the resolution of visual perception, the electrodes need to be made as small as possible, while being capable of delivering an effective level of charge safely. Finite element modeling has suggested that altering annulus height will alter stimulus efficacy (Brunton et al., 2012), but this has yet to be confirmed with *in vivo* comparisons of the stimulation efficacy of electrodes with different annulus heights, to ensure the electrode area can be made as small as possible while keeping the charge densities required to be effective, within “safe” limits.

Another alteration to improve the safety of stimulation of the penetrating microelectrodes is the addition of an electrode coating to improve the CIC of the electrode. Coating electrodes with a thin film of titanium nitride (TiN) increases their CIC *in vitro* (Janders et al., 1996; Zhou and Greenberg, 2003). TiN has already been used in cardiac pacemakers, cardiac valves and orthopedic prostheses (Zhou and Greenberg, 2003; Cogan, 2008) and has the advantage that it delivers charge mainly through capacitive means, which is desirable as no unwanted substances are created or consumed via Faradaic reactions at the electrode tissue interface (Merrill et al., 2005; Cogan, 2008). While TiN has been studied extensively in the *in vitro* environment (Janders et al., 1996; Weiland et al., 2002; Zhou and Greenberg, 2003), there is considerably less information about how TiN coated electrodes will perform in the *in vivo* environment, especially in the cortex and whether the TiN coatings survives the penetration into cortical tissue *in vivo*. Furthermore, as there is a large variability between methods of calculating charge injection limits between different research groups, it is important to compare the CIC of different electrodes under the same conditions (Venkatraman et al., 2011).

In this study we have investigated the charge densities per phase required to evoke motor response in the anesthetized rat using annular electrodes with varying geometric surface areas (GSAs). GSAs were altered by varying the height of the annular electrode contact from 20 to 100 μm, resulting in GSAs ranging from 7000 to 41000 μm^2^ for a 125-μm diameter electrode. Annulus height was varied rather than the diameter of the electrode shaft, as this is an easy alteration to make at the final stages of the electrode manufacturing process. We recorded the charge density per phase required to evoke a motor response (movement of the large face whiskers – “whisking”) to stimulation. Furthermore, we compared the *in vivo* CIC of PtIr electrodes with and without a coating of sputtered TiN. This allowed us to determine the minimum GSAs required for the electrodes with and without a TiN coating in order to ensure stimulation will be both effective and safe using our annulus electrodes.

## Materials and Methods

### Electrode Fabrication

Electrodes were manufactured by partners MiniFAB (Melbourne, VIC, Australia) and were all platinum/iridium (PtIr, 20% iridium) electrodes insulated in Parylene C. As described elsewhere (Brunton et al., 2012; Wang et al., 2013), laser ablation was used to remove the insulation from an annular region around the electrode shaft, to a height of 20, 40, 70, or 100 μm, so as to compare the effect of varying the electrode’s GSA. Henceforth these electrodes will be referred to as uncoated PtIr electrodes. (Note that all electrodes were insulated with Parylene C and our use of the term coated or uncoated refers only to whether TiN was additionally sputter coated onto the PtIr electrodes).

**FIGURE 1:**
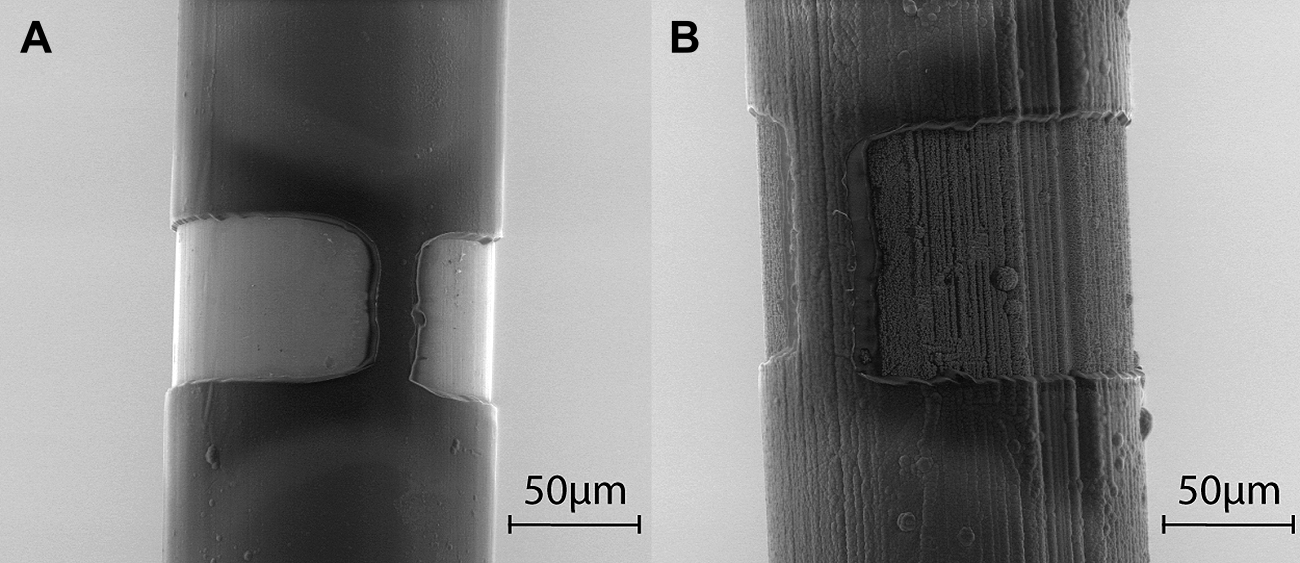
**Scanning electron microscope images of annuli. (A)** Uncoated PtIr electrode with 70 μm annulus. **(B)** TiN coated electrode with 100 μm annulus. Due to shadowing, some portion of the electrode cannot be ablated completely, resulting in an incomplete ring around the electrode, thus the geometric surface area (GSA) is less than if the electrode contact was a complete ring.

To develop the TiN-coated PtIr electrodes (henceforth referred to as TiN-coated electrodes), TiN was first sputter-coated onto the bare PtIr electrodes before they were coated with Parylene C and laser ablated (**Figure 1**). Previously optical microscopy has been used to measure the GSAs of the electrodes with annulus heights from 40 to 100 μm and the average surface area is listed in **Table 1**. The surface area of the electrodes with 20-μm annuli was assumed to be half that of the 40 μm annuli as the surface area of these electrodes was difficult to measure under optical microscopy due to the small annulus height. Due to the extra thickness of the TiN coating, the diameter of these electrodes was approximately 20 μm larger than the uncoated PtIr electrodes. This resulted in the TiN-coated having a larger electrode GSA for the same annulus height when compared to the PtIr electrodes. **Figure 1** shows the morphology of the two different electrode materials under scanning electron microscopy; note that the TiN electrodes (**Figure 1B**) have a highly porous structure resulting in a much larger real surface area (RSA) when compared to the uncoated PtIr electrodes.

**Table 1:** Geometric surface area (GSA) of electrodes.

Material	Annulus Height (μm)	Geometric Surface Area (× 10^-4^ cm^2^)
PtIr	40 70 100	1.4 2.3 3.8
TiN	40 70 100	1.6 2.4 4.1

*The GSA of the electrodes was measured under optical microscopy the mean GSA for each annulus height is shown.*

### Surgical Procedure for *In Vivo* Experiments

All animal care and experimental procedures complied with the Australian Code of Practice for the Care and Use of Animals for Scientific Purposes and were approved by Animal Ethics Committee of Monash University. Ten Sprague Dawley rats, weighing 350–500 g, were used for this study. The general procedures for electrical stimulation using our annulus electrodes in the rat motor cortex have been described previously (Wang et al., 2013) and general surgical procedures conformed to our other previous reports (Alwis et al., 2012; Johnstone et al., 2013). Anesthesia was induced in a closed box with 5% halothane in O_2_. Once deep anesthesia was achieved, with absence of the noxious pinch withdrawal reflex, the animal was taken out of the box and attached to a nose cone fed with halothane and a tracheotomy was performed to insert and secure a tracheal cannula. The tracheal cannula was connected to the anesthetic delivery respirator and the animal was switched to maintenance dosages of halothane (1.5–2%) with 0.3 ml/min O_2_ for further surgery and experimentation. The electrocardiogram and electromyogram were monitored continuously via clip electrodes placed on the forepaw upper musculature (Alwis et al., 2012; Johnstone et al., 2013). Body temperature was maintained at 37.5° via a thermostatically regulated thermal pad with feedback from a rectal probe (Fine Science Tools Inc., Canada). The animal’s skull was cleared of skin and periosteum and the head was secured in place via a head bar held in a magnetic stand; the head bar was anchored to the skull via a bone screw placed above the left hemisphere near occipital cortex, and the whole assembly of bar and screw was further secured with dental acrylic.

A 304 grade stainless steel screw was secured in the skull to contact the Dura mater, a few mm anterior to the craniotomy, to act as a non-current carrying reference electrode. Stainless steel screws have a stable reference potential and have commonly been used as reference electrodes *in vivo* as an alternative to Ag/AgCl (Weiland and Anderson, 2000). For a current sink (counter electrode) a large surface area platinum wire was placed in the temporalis muscle and secured with super glue.

**FIGURE 2:**
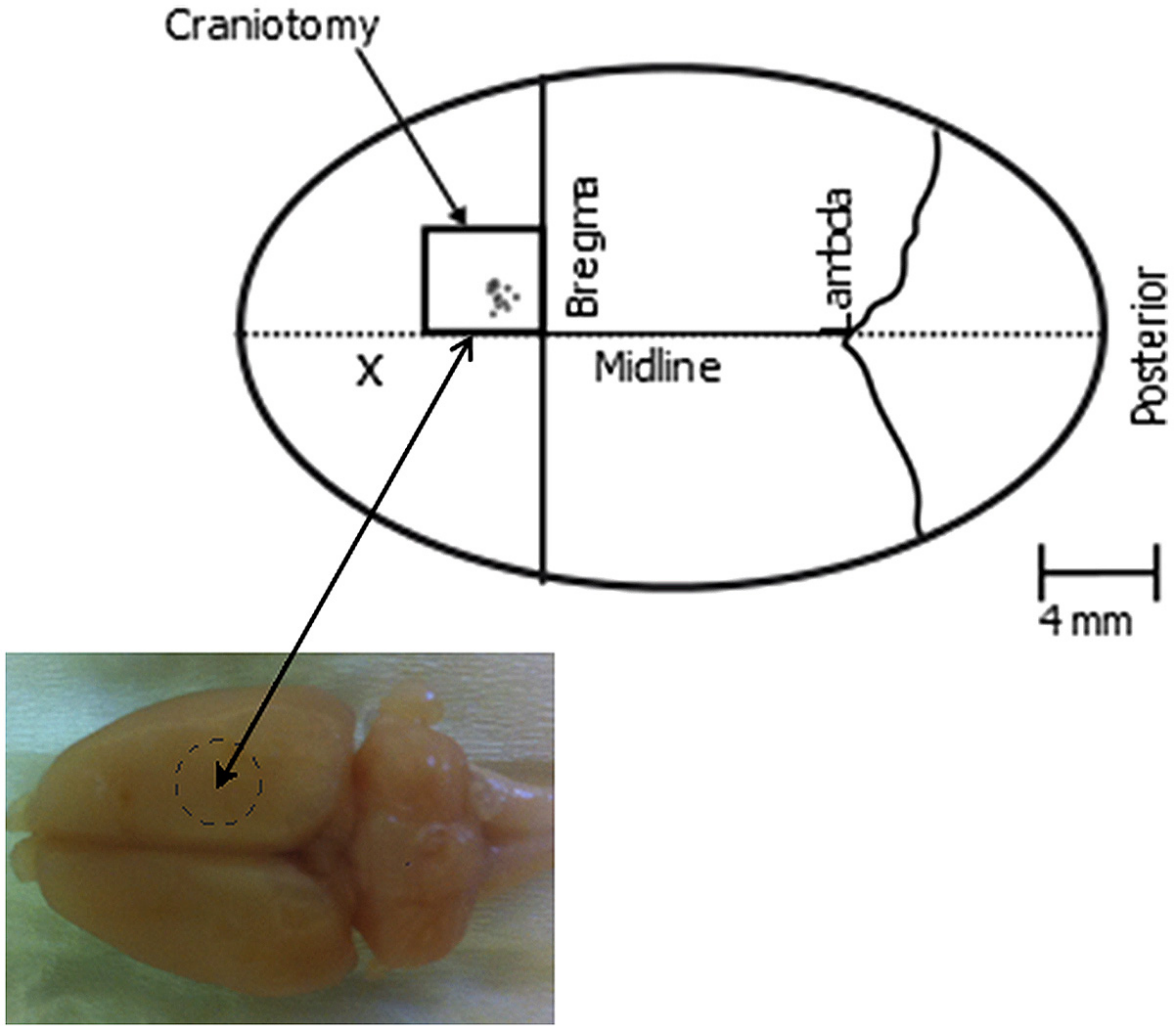
**Schematic of the implantation site.** The box indicates the region of the craniotomy which was approximately 4 by 4 mm. The individual electrode placements used are indicated by circles within the craniotomy region. X denotes the approximate position of the stainless steel screw.

A 4 mm × 4 mm craniotomy was made over the right hemisphere to expose the primary motor cortex (**Figure 2**). The Dura mater was carefully cut from above this region of cortex and peeled back, to allow the microelectrodes to directly contact and then enter the cortical tissue.

### Charge Injection Capacity

**FIGURE 3:**
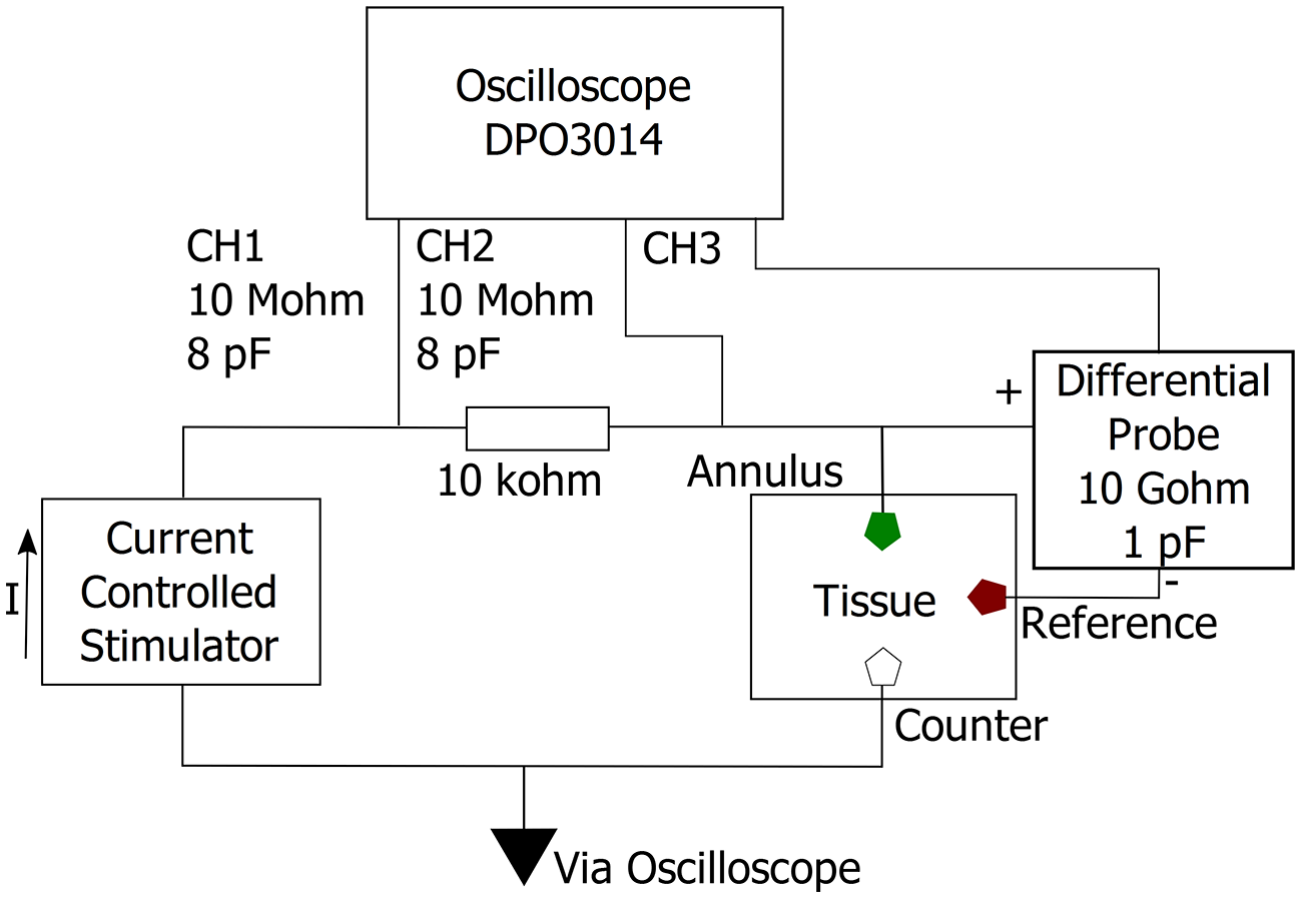
**Measurement circuit.** The voltage response to a symmetric biphasic current pulse was measured between the annulus electrode (working) and a non-current carrying stainless steel screw (reference). Current was measured by measuring the voltage drop over a 10 kΩ resistor connected in series with the working electrode.

Charge injection capacity was measured both *in vitro* and *in vivo*. Ten electrodes, five uncoated PtIr, and five TiN coated, were used to measure the CIC of the electrodes. The *in vitro* measurements were conducted in 0.1 M phosphate buffered saline (PBS) using a three electrode setup, with a platinum wire counter and Ag/AgCl reference electrode. For *in vivo* measurements, the electrodes were implanted so that the annulus was located at a depth of approximately 1500 μm. All measurements were taken in the same animal. A stainless steel screw was used as the reference electrode, as an alternative to the Ag/AgCl electrode, and the same platinum wire as used *in vitro* was used as a counter electrode *in vivo*. The voltage between the working and reference electrode in response to a single symmetric biphasic current pulse was measured via a custom-made differential amplifier connected to a DPO3014 oscilloscope (Tektronix). The differential amplifier was made using an OPA445 operational amplifier (Texas Instruments) with a resultant input impedance of approximately 10 GΩ in parallel with one pF. The electrodes were unbiased and allowed to return to their open circuit potential when not pulsing. Symmetric biphasic current pulses were delivered to the working electrode using an isolated current source (Digitimer Model: DS4). Current amplitudes and pulse durations were programmed using a custom script developed in Spike2 software (Cambridge Electronic Design, CED, UK). Stimulus parameters were entered via a graphic user interface and were used to generate voltage pulses at a digital to analog output port available on a CED 1401 system. The Digitimer current source was gated and controlled after receiving voltage pulses from the CED 1401 device. The delivered current was determined by measuring the voltage across a 10 kΩ resistor that was connected in series with the working electrode (**Figure 3**). Biphasic current pulses with pulse widths of 100 μs and interphase gaps of 50 μs were used for measuring CIC.

**FIGURE 4:**
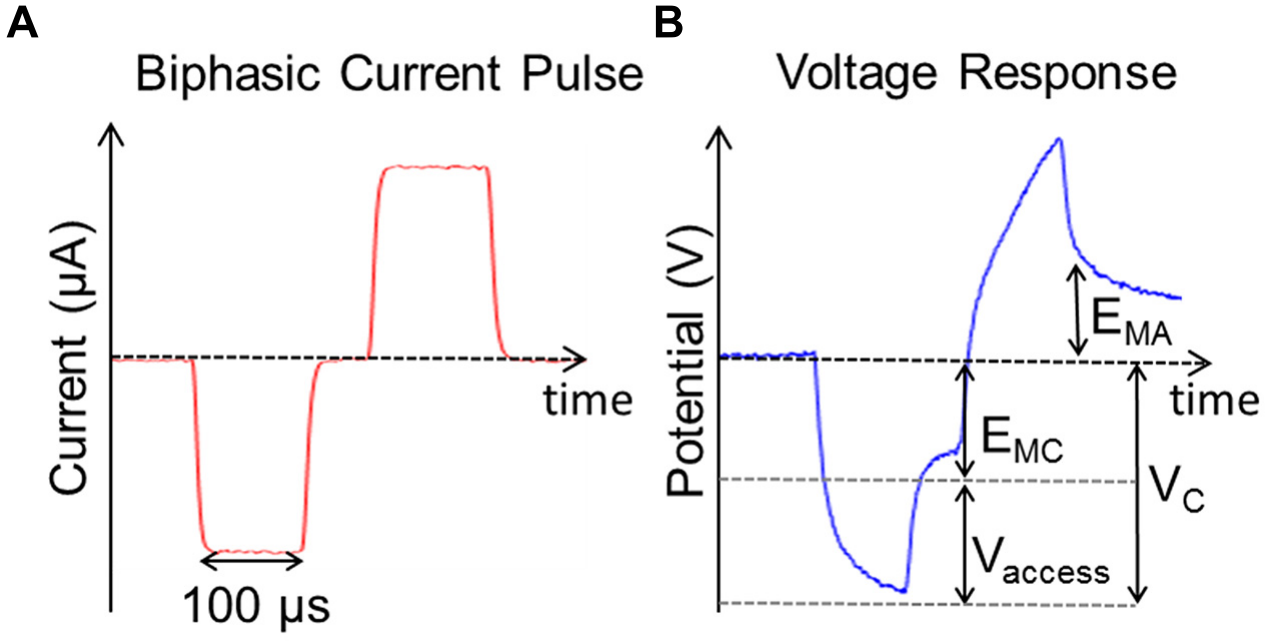
**Stimulation used to measure charge injection capacity. (A)** Single symmetric biphasic current pulse measured between CH1 and CH2. **(B)** Voltage response to current pulse shown in **(A)** where E_MC_ is the negative polarization voltage, E_MA_ is the positive polarization voltage, V_C_ is the peak cathodic voltage, and V_access_ is the voltage drop across the tissue. The voltage is measured referenced to a stainless still screw and then shifted to be referenced to Ag/AgCl.

The occurrence of unwanted Faradaic reactions such as water electrolysis can be minimized by keeping both the positive and negative maximum potentials across the electrode-tissue interface within a defined window (Rose and Robblee, 1990). Safe charge injection limits were defined by the charge that could be injected in a single phase of the current pulse before either *E_MC_* or *E_MA_* exceeded the window for water electrolysis which is -0.6 to 0.8 V versus Ag/AgCl for PtIr (see **Figure 4**). *E_MC_* and *E_MA_* were defined as the voltage measured 20 μs after the end of the cathodic and anodic phases of the current pulse, respectively. The voltage was normalized for the stainless steel reference. The charge injected was then divided by the electrode’s GSA to give the CIC as in (Tykocinski et al., 2005; Leung et al., 2014). Though the water window is wider for TiN than that of PtIr, CIC was determined for both electrodes based on the water window of PtIr, as the TiN was sputter coated onto the surface of the PtIr electrodes; it was possible some PtIr would still be exposed to the tissue.

### Testing for *In Vivo* Stimulus Efficacy

Electrodes were attached to a high speed microelectrode controller (Kopf Model 2660) mounted on a complex of translators and goniometers (Alwis et al., 2012; Johnstone et al., 2013) and inserted perpendicular to the cortical surface, at a point approximately between 0.9 and 2 mm lateral from midline and 1.4 and 2.2 mm anterior from Bregma (**Figure 2**), a region corresponding to the region of motor cortex that can be stimulated to evoke movement of the whiskers (Brecht et al., 2004; Cramer and Keller, 2006; Wang et al., 2013). High power surgical microscopy was used to visualize contact of the electrodes with the cortical surface, and the microdrive was zeroed at this point to ensure high precision in determining the electrode position within the brain during further advancement to find the optimal depth for evoking whisker movement. Current pulses were used to evoke movement of the whiskers, with a stainless still clip placed on the neck muscle being used as the current return for all stimulation trials.

Using the high-speed microdrive, the electrode was advanced from the cortical surface until the electrode tip was positioned at a depth between 1.8 and 2.2 mm. This located the annulus at a depth of 1.2 to 1.6 mm, a depth previously shown to be effective when stimulated to evoke motor responses in anesthetized rats (Tandon et al., 2008). For each individual animal, several cortical penetrations were made with the electrode until a location was found where strong whisker movements were evoked with stimulation at 100 μA. All electrode positions were referenced to the Midline and Bregma and the depth of the electrode tip (and hence the annulus) from the cortical surface was always noted. All subsequent electrode penetrations, to test other electrode types or annulus sizes, were made to position the electrode annulus as close as possible to this optimal position.

**FIGURE 5:**
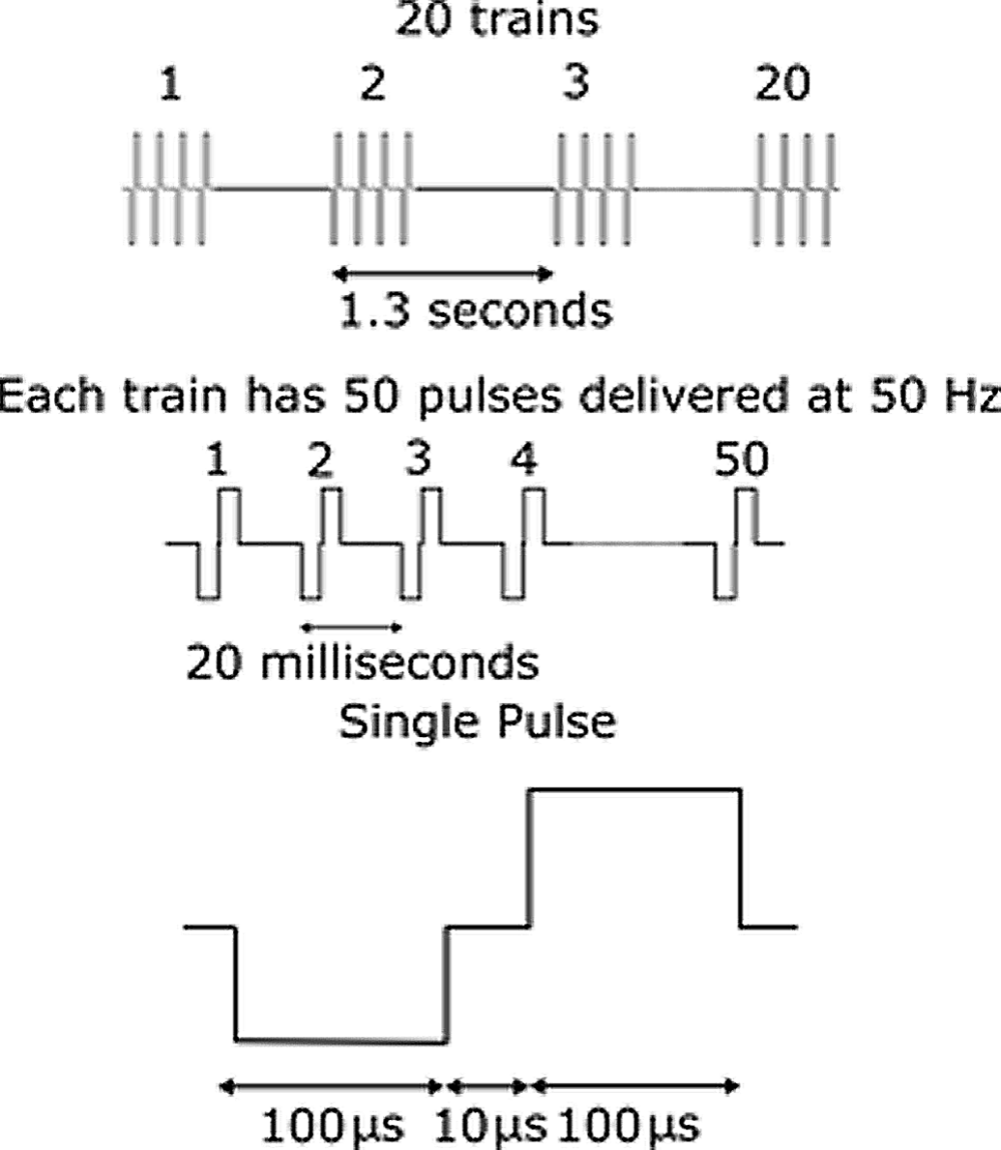
**Illustration of stimulation paradigm.** Current amplitude was varied while all other parameters were held constant. 20 trains of 50 symmetric biphasic cathodic first current pulses, frequency 50 Hz, pulse width 100 μs, interphase gap 10 μs at intervals of 1.3 s were delivered via a custom designed stimulator system.

To evoke whisker movement, 20 trains of 50 symmetric biphasic cathodic first current pulses, frequency 50 Hz, pulse width 100 μs, interphase gap 10 μs at intervals of 1.3 s were delivered via a custom designed stimulator system. The stimulation paradigm is illustrated in **Figure 5**. We have previously found that this stimulation regime reliably evokes whisker movements in awake animals (Wang et al., 2013). The current amplitude could be adjusted to any value between 2 and 100 μA. The stimulating electrode was shorted to the return electrode after every pulse train for 300 μs, to avoid charge build up on the electrode surface.

**FIGURE 6:**
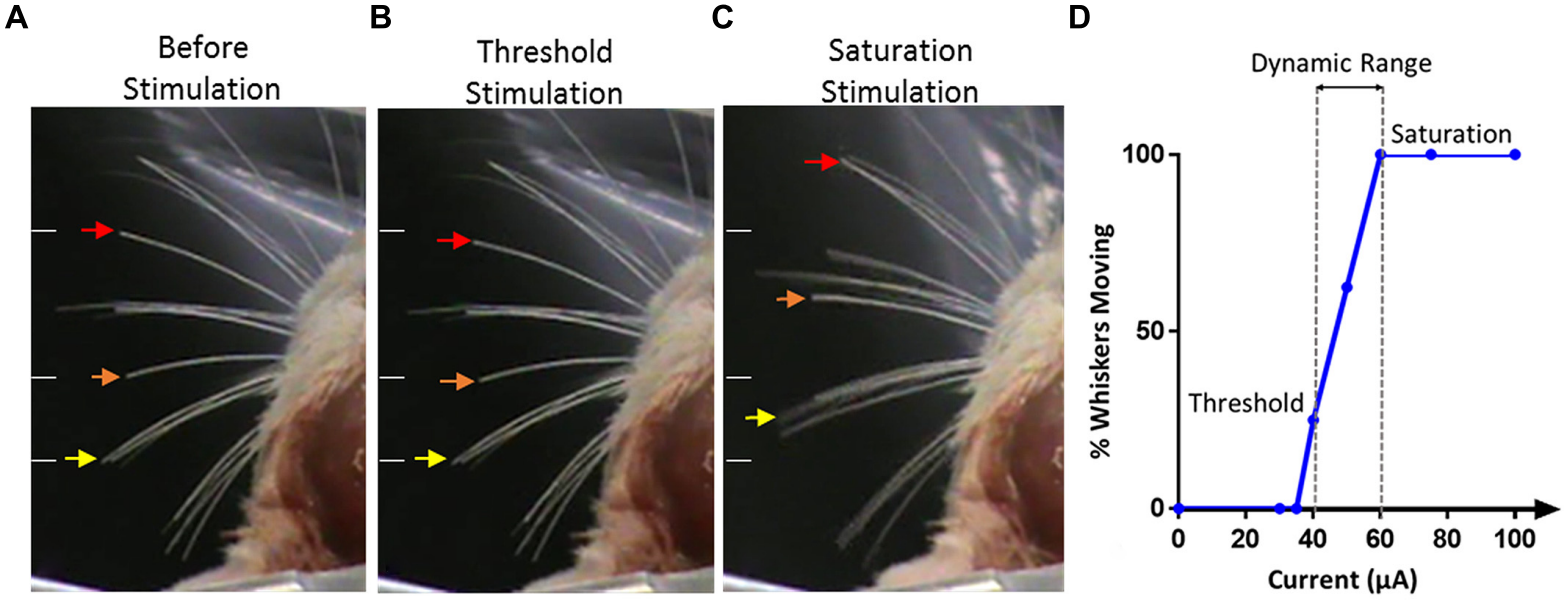
**Stimulation evoked whiskers movement. (A)** The whiskers are in their initial positions before stimulation. Arrows indicate the movement of three whiskers of interest. White lines on the left of each panel indicate the initial position of the whiskers and are kept in the same position across all panels for reference. **(B)** Stimulation applied at threshold, only two whiskers, as identified by the orange and red arrows, were seen to move from their resting positions. **(C)** With saturated stimulation, many whiskers moved in either anterior or posterior direction from the resting points. Saturated stimulation also evoked whiskers movement on the ipsilateral side of stimulation which is not shown in this figure. **(D)** An example of the response from one electrode illustrating the percentage of whiskers moving versus current amplitude, threshold, saturation, and dynamic range are identified.

The threshold current for stimulation was defined as the current level where visually detected whisker movements occurred in two out of three consecutive trials, with three expert observers being involved in all observations, independently. Video recordings were also taken for oﬄine confirmation of whisker movement evoked by electrical stimulation. The response was graded by counting the number of whiskers active in response to a given stimulation current. The saturation current was determined as the current where a further increase in current elicited no further increase in the visually observable response either in the number of active whiskers or the size of the whisker movement elicited; if the maximum current (100 μA) that could be delivered by the stimulator was reached before this saturation response occurred, then the maximum current was considered to be the saturation current. The difference between the threshold current and saturation current was termed the ‘dynamic range,’ the range over which a variation in functional effects was seen. **Figure 6** shows a typical movement at threshold, where only two whiskers were found to respond to the stimulation at threshold and at saturation were a large number of whiskers were seen to move on both the contralateral and ipsilateral sides.

## Results

### Charge Injection Capacity

The measured CIC was determined from the charge that could be injected before the potential exceeded the water window of PtIr when stimulating with single biphasic current pulses. Values of CIC for the different electrodes tested are listed in **Table 2**. All electrodes coated with TiN had larger CIC than the uncoated PtIr electrodes. The average CIC of the TiN coated electrodes was 72 μC.cm^-2^.ph^-1^ compared with the average CIC of PtIr of 18 μC.cm^-2^.ph^-1^. In all cases *E_MC_* was exceeded before *E_MA_*.

**Table 2:** Charge injection capacity (CIC).

Material	*In vitro* CIC (μC.cm^-2^.ph^-1^)	*In vivo* CIC (μC.cm^-2^.ph^-1^)
PtIr	23 5	18 5
TiN	194 17	72 13

*The CIC of each of the electrodes was measured using single biphasic symmetric current pulses with pulse widths of 100 s. The results are presented as mean SD.*

**FIGURE 7:**
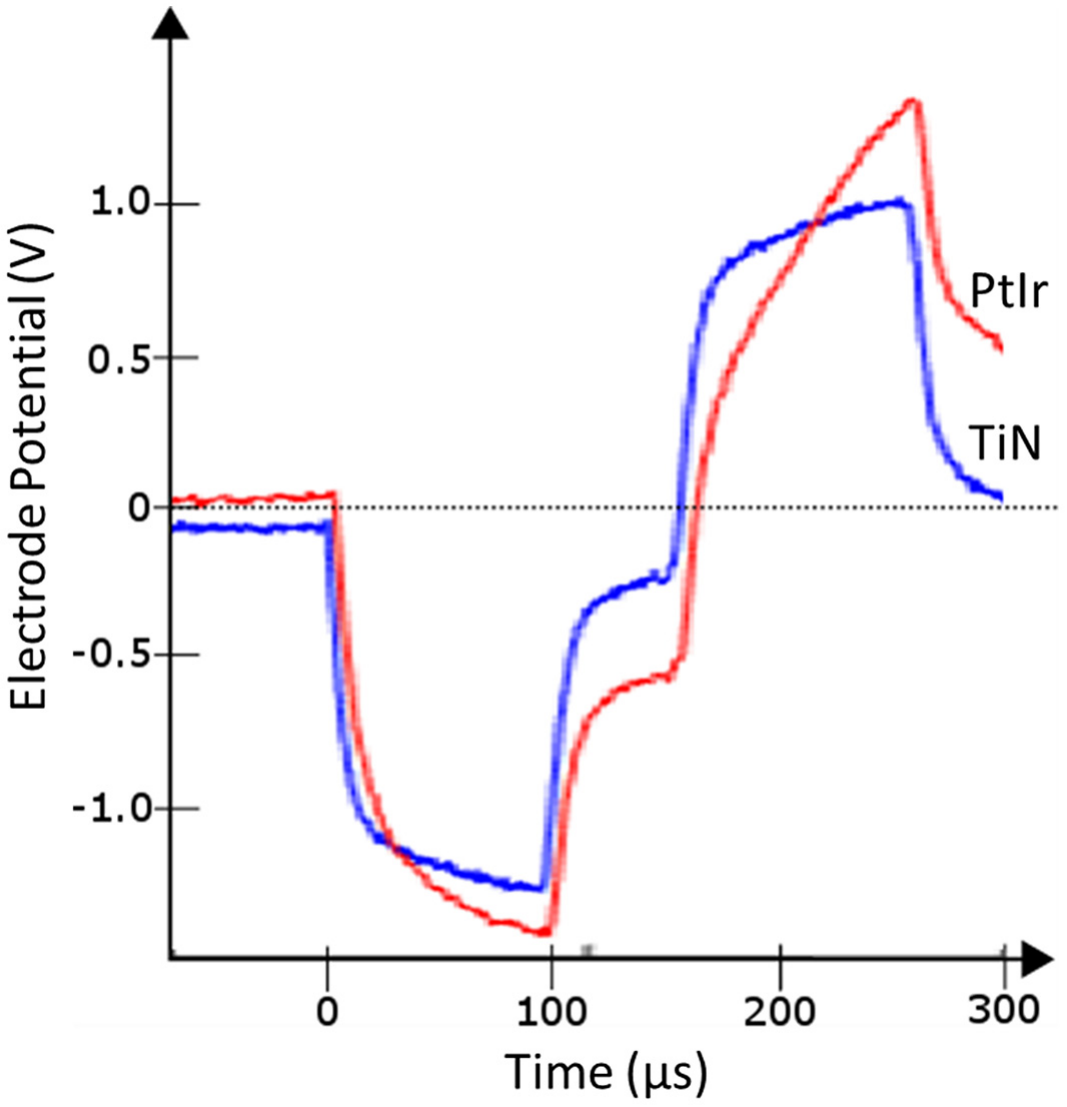
***In vivo* voltage response to a 50 μA, 100 μs per phase biphasic current pulse from electrodes with 40 μm annuli, TiN coated electrode (blue line), and uncoated PtIr electrode (red line)**.

**Figure 7** compares the voltage response of TiN and PtIr electrodes with 40-μm annuli, to a 50 μA, 100 μs per phase biphasic current pulse, with interphase interval of 50 μs. This equates to a charge per phase of 5 nC. *E_MC_* across the uncoated PtIr electrode was measured to be -0.68 V compared with -0.37 V on the TiN coated electrode. With charge injection of 5 nC corresponding to a charge density of 35.7 and 31.3 μC.cm^-2^.ph^-1^, for the uncoated PtIr electrode and TiN coated electrode, respectively, the potential drop across the PtIr electrode has already exceeded the water window, whereas the potential drop across the TiN coated electrode is still well within the water window.

### *In Vivo* Stimulation

Whisker movements were monitored in response to current stimulation of motor cortex and thresholds were defined as the lowest current level where whisker movements were reliably observed. In total, 21 electrodes were tested in 10 animals, eight TiN coated electrodes, and 13 uncoated PtIr electrodes. Up to four electrodes were tested in the same animal and in seven out of the 10 animals two or more electrodes were used for threshold measurements.

**FIGURE 8:**
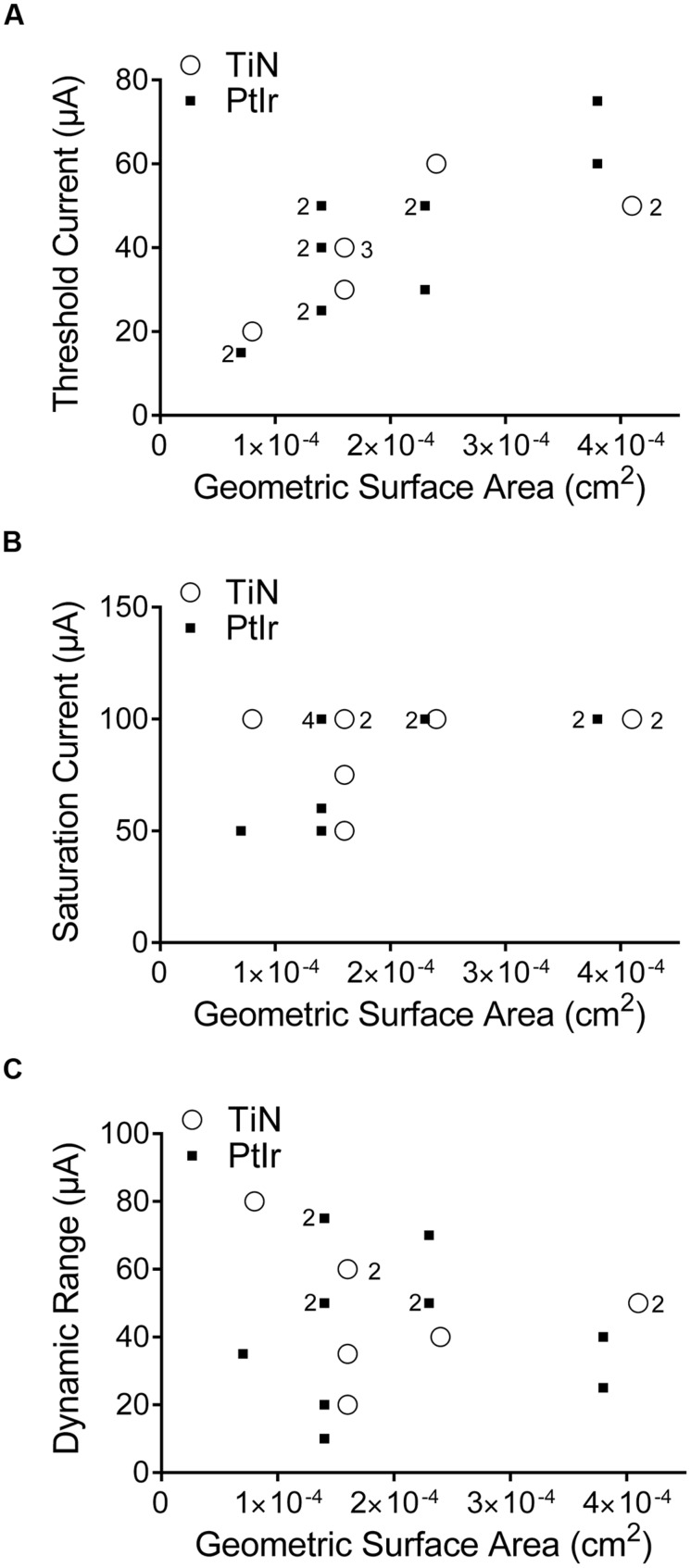
**Stimulation currents used versus the geometric surface area of the electrode. (A)** Threshold current, **(B)** saturation current, and **(C)** dynamic range. TiN coated electrodes are indicated by open circles and uncoated PtIr electrodes are indicated by solid squares. The numbers to the left of a marker indicates the corresponding number of PtIr overlapping data points, the numbers to the right of a marker indicates the corresponding number of TiN overlapping data points. No number indicates only one electrode.

The threshold current versus GSA for all electrodes is shown in **Figure 8A**. As GSA increased, the current amplitude required to evoke a whisking response also increased, for both electrodes types. In order to determine whether there was a statistically significant difference between the thresholds of the two electrode types, the slopes, and intercepts of the two data sets were compared using GraphPad Prism 6 (GraphPad Software, San Diego, CA, USA). No statistical significance between the uncoated PtIr and TiN coated electrodes were found with *p*-values of 0.12 and 0.55 for the slopes and intercepts, respectively.

The saturation current was defined as the current amplitude where a further increase in current resulted in no further increase in the observed functional response. Saturation current was determined for all eight TiN-coated electrodes and all but one of the 20 μm uncoated PtIr electrodes. **Figure 8B** shows the saturation current versus GSA; there was no correlation between GSA and saturation current. It must be noted that the current limit of the ratpack stimulator was 100 μA and that it was possible that some electrodes would have saturated at current amplitudes greater than 100 μA, but due to the limit on the stimulator no greater increase in current amplitude could be tested; however, at 100 μA all electrodes showed large-amplitude bilateral whisker movements. Again a comparison of the slopes and intercepts found no statistical significance between the two electrode types (*p* = 0.46 and 0.97 for the slopes and intercepts, respectively).

Finally, the dynamic range of the electrodes is shown in **Figure 8C**. The dynamic range is defined as the saturation current minus the threshold current. There was a large variation in the dynamic range, even amongst the same electrode type and annulus height, but there was no correlation between GSA and dynamic range.

**FIGURE 9:**
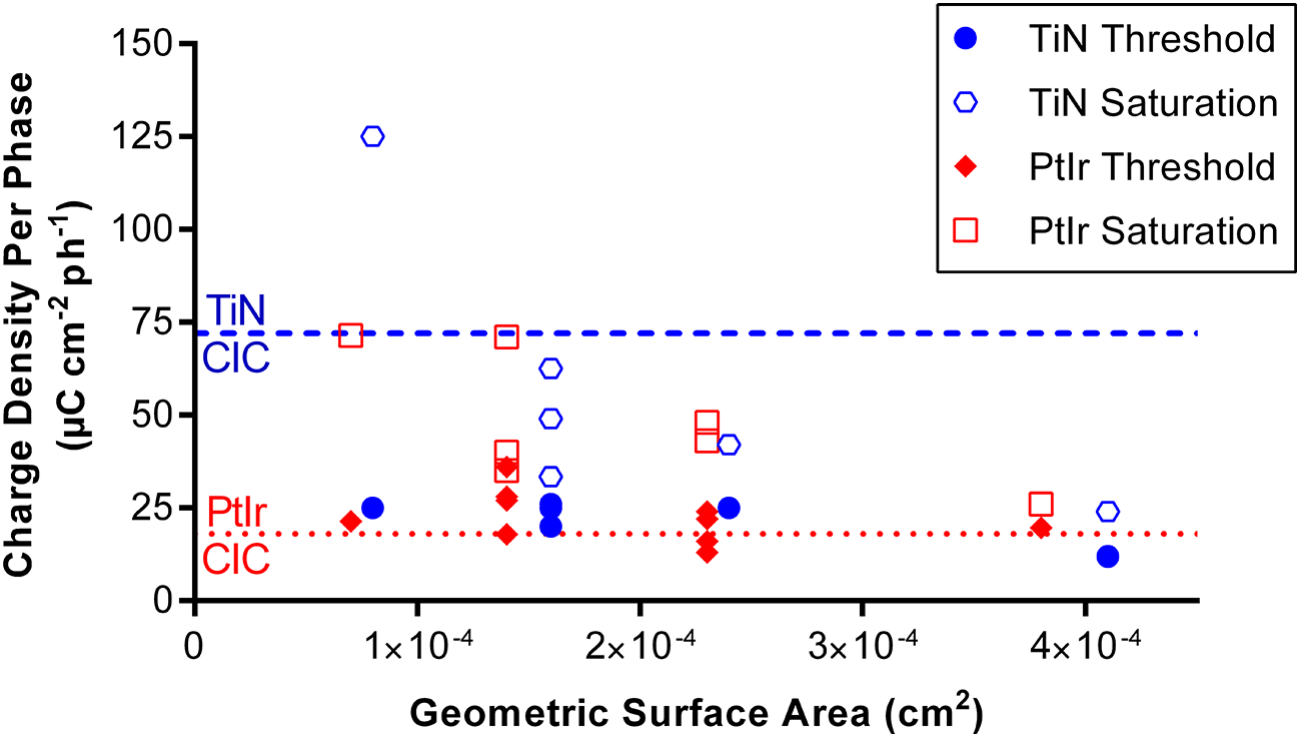
**Threshold and saturation charge densities per phase plotted against the geometric surface area of the electrode.** Dash blue line indicates the mean CIC of the TiN coated electrodes. Dotted red line indicates the mean CIC of the uncoated PtIr electrodes.

**Figure 9** plots the threshold and saturation charge densities per phase as a function of GSA compared with the average CIC of each electrode type. It can be seen that all of the stimulation charge densities used on electrodes with GSAs greater than 14,000 μm^2^ in this study fall below the average CIC measured from the TiN coated electrodes. By comparison, many of the charge densities used for stimulation are above the CIC measured from the uncoated PtIr electrodes. In particular many of the threshold charge densities are above the CIC of the uncoated PtIr electrodes.

## Discussion

This study had two main objectives: first, to determine the charge densities per phase required to evoke a motor output (i.e., movement) when stimulating with novel annulus electrodes with different annulus heights; second, to determine the *in vivo* CIC of PtIr electrodes with and without a coating of porous TiN. This information allowed us to determine the GSAs of the electrodes required to ensure safe stimulation using PtIr electrodes with and without a coating of porous TiN.

### Charge Injection Capacity

In regards to the safety of stimulation, it is preferable that any charge transferred into the tissue should occur via either non-Faradaic or reversible Faradaic reactions (Brummer and Turner, 1975). The voltage drop across the electrode-tissue interface (*E_MC_*) governs the electrochemical reactions that occur at the interface (Brummer et al., 1983). Water electrolysis should be avoided during stimulation as they result in the formation of oxygen and hydrogen gasses, which quickly diffuse away from the electrode surface into the tissue (Brummer and Turner, 1975). The occurrence of these reactions can be minimized by keeping the potential across the electrode within a defined potential window, the “water window,” which is specific to the electrode material used. Here, we have determined the CIC of the electrodes from the maximum charge that could be injected before *E_MC_* exceeded the water window of PtIr when stimulating with single biphasic current pulses. Though TiN has a wider water window, we chose to measure the CIC of the TiN coated electrodes based on the water window of PtIr as it was possible, although unlikely, that the TiN coating may not have completely covered the PtIr underneath. Additionally we only considered the CIC measured from symmetric biphasic pulses and it has been shown that the use of asymmetric, biased current pulses can significantly improve the CIC of electrodes (Cogan et al., 2006).

The *in vitro* CIC measured here is less than what has been seen in previous studies using voltage transients, 100–150 μC.cm^-2^.ph^-1^ for PtIr electrodes (Rose and Robblee, 1990) and approximately 860 μC.cm^-2^.ph^-1^ for TiN electrodes (Weiland et al., 2002). Here, we have used shorter pulse widths to measure CIC than these previous studies. The use of shorter pulse widths has been shown to reduce CIC (Leung et al., 2014). The use of different setups and methods will also greatly influence the measured CIC (Venkatraman et al., 2011). The CIC of PtIr electrodes measured here *in vitro* is similar to those found by others (Leung et al., 2014) using Pt disk macroelectrodes, using a similar method to that used here. The CIC of both electrodes was reduced *in vivo* compared to *in vitro*. The reduction of CIC *in vivo* is consistent with studies of AIROF microelectrodes that have shown CIC to significantly reduce *in vivo* due to increased electrode polarization voltages for the same applied charge (Hu et al., 2006). This reduction in CIC is expected as the *in vivo* environment is significantly different to the *in vitro* environment; *in vivo* there is an unknown concentration of ions, and an inflammation response that impedes the flow of charge (Polikov et al., 2005; Cogan, 2006, 2008). Cell adsorption onto the surface of electrodes may also reduce their functional surface area, thus increasing their impedance and reducing their capacitance, in turn increasing the voltage required to inject the same current for the same period of time. In (Musa et al., 2009) it was shown that the presence of bovine serum albium in the electrolyte significantly reduced the charge density at which gas evolution occurred. Previously we have reported on impedance changes in chronic *in vivo* stimulation studies (Wang et al., 2013). We found that there were two phases of impedance change that occurred post-implantation, an initial significant increase in impedance during the first 6 weeks following implantation, followed by a slower and more-gradual impedance decrease to values similar to those seen in the immediate post-implantation stage. These impedance changes were suggested to be due to remodeling of the surrounding neural tissue post-implantation due to the foreign body response. Furthermore, we found that following short bursts of stimulation, an electrode’s impedance could dramatically reduce (Wang et al., 2013). These changes in the electrode tissue interface with time, and following stimulation, will likely influence the CIC of the electrodes and measurements of CIC in chronic implantations are needed to ensure that it does not significantly reduce over time. Nevertheless, we have demonstrated that PtIr electrodes coated with TiN still show an increased CIC compared to the uncoated PtIr electrodes in the *in vivo* environment.

Another consideration is that CIC as measured via voltage transients underestimates the charge that can be delivered without causing significant tissue damage as shown by histopathological studies (Leung et al., 2014). This is reflected in that the CIC measured here for uncoated PtIr electrodes is well below the value of 52 μC.cm^-2^.ph^-1^ which has been shown to be safe, using histological criteria, with long term stimulation of Pt electrodes (Ni et al., 1992). Larger electrode polarization potentials are tolerable under pulsed current stimulation before gas evolution occurs, due to the high frequency of stimulation pulses (Musa et al., 2009). It is also likely that biological tissue can cope with low rates of water electrolysis without significant damage (Leung et al., 2014). Overall, electrochemical studies need to be combined with histology to determine true safety limits of these electrodes and the long term tissue damage that might occur when the water window is broken.

### Stimulation Efficacy: Chronic versus Acute

The charge density per phase required to evoke stimulation here was less than we found in chronic implantations studies (Wang et al., 2013; Brunton et al., 2015). This was unexpected as anesthesia reduces cortical responsiveness and hence it was expected that a larger charge density per phase would be required to elicit a motor response to stimulation. However, in this experiment there was a large degree of freedom as to where the electrode annulus could be positioned within the cortex, and the electrodes were moved to a position where maximum effects of the stimulation were seen. In contrast, in our previous chronic experiments (Wang et al., 2013; Brunton et al., 2015) an electrode array is implanted, thus we were not able to position each electrode in the array individually, as the electrode annuli were located at a fixed position from the array cap. The position of the array in these previous studies was within a similar window within the cortex corresponding to the whisker motor cortex, but the position of each of the electrodes was constrained by the need to minimize the likelihood of piercing large surface blood vessels that could result in hemorrhage, rather than where stimulation would be most effective. Another possible reason for the increase in chronic thresholds may be the cell death and the foreign body inflammation response that results in fibrous capsule formation around the array. This fibrous capsule may push neurons further away from the electrode, increasing the required threshold current (Polikov et al., 2005).

### Stimulation Efficacy: Electrode Material

We found no difference in terms of stimulation efficacy between the uncoated PtIr electrodes and the TiN coated PtIr electrodes. The threshold and saturation currents versus GSA were similar for the two electrode types (**Figure 8**) despite TiN coated electrodes having a much rougher surface and thus a greater RSA than the uncoated PtIr electrodes. This indicates that the electrode’s GSA has a greater influence on an electrode’s efficacy than its RSA.

### Stimulation Efficacy: Geometric Surface Area

For larger GSAs, the current required to evoke whisker movements was also higher. Previous studies have shown similar results (Bagshaw and Evans, 1976; West and Wolstencroft, 1983) and this difference in regards to electrode GSA was also expected from our previous modeling work (Brunton et al., 2012, 2013). Neural excitation patterns elicited from external stimulation via axons can be predicted by the activating function, *f*, which is proportional to the second spatial derivative of the extracellular potential (Rattay, 1989, 1999). Electrodes that have smaller GSAs have a greater variation in the electric field in regions close to the electrode, with larger values of *f* than electrodes with large GSAs. Thus it is more likely that a small GSA electrode will activate axons in this region with a lower current than that required with a large GSA electrode. Using the activating function to determine the likelihood of neural stimulation also predicted that electrodes with a larger GSA would have a narrower dynamic range, i.e., the current range that produces a change in functional response (Brunton et al., 2012). When higher currents are used, such as those at saturation levels, the electric fields further away from the electrode may become sufficient to evoke stimulation. In these regions, the field is not influenced by the exact GSA of the electrode. Thus, the current levels required to see saturation of an evoked response are similar regardless of the GSA of the electrode (Brunton et al., 2012). However, in this study we were not able to precisely determine saturation current levels as the stimulator could only deliver up to 100 μA. While all electrodes showed strong bilateral whisker movements at 100 μA, because the current could not be increased beyond this level, we do not know if a further increase in current would have resulted in further increase in response.

### Choosing Electrode Geometric Surface Area

The fact that as GSA increased, the threshold charge density per phase decreased, has important implications in the selection of the electrode’s GSA. Electrodes with smaller surface areas require lower currents to activate neurons but the electrodes need to be large enough so that the charge density required for stimulation is within an electrochemically “safe” level, i.e., the water window (Brummer et al., 1983). Here, we have considered this safe level as the charge that can be injected before the potential across the electrode exceeded the window for water electrolysis. While this method is conservative and underestimates the charge that can be injected before tissue damage is observed, it guarantees the absence of water electrolysis (Rose and Robblee, 1990). The CIC is material dependent, with the CIC of electrodes coated with TiN being more than three times greater than the bare PtIr electrodes. We can use the CIC measured here to determine a conservative minimum electrode GSA. From **Figure 9** we can see that even for the electrodes with the largest GSAs, the threshold charge densities required for effective stimulation in this study, are very close to the CIC measured for PtIr, thus if PtIr is to be used in a cortical prosthesis it is recommended that the surface area of these electrodes is greater than 38,000 μm^2^ to minimize the likelihood of water electrolysis. By comparison, if the electrodes were coated with TiN, the effective charge densities per phase required for stimulation using electrodes with GSAs of 16,000 μm^2^, would result in electrode polarization voltages within the water window. Furthermore, the CIC of the electrodes can be increased by altering the stimulation paradigm, such as adding a biasing potential (Cogan et al., 2006). If these stimulation paradigms were implemented, electrodes with smaller GSAs could be used.

As mentioned above, measuring an electrode’s CIC via voltage transients provides conservative safety limits. If instead we consider the limit of 52 μC.cm^-2^.ph^-1^ shown to be safe by histological analysis with chronic stimulation of the auditory nerve in cats (Ni et al., 1992), a less conservative minimum GSA of 23,000 μm^2^ corresponding to an annulus height of 70 μm could be used for the PtIr electrodes.

Future work is needed to investigate the histological damage associated with long periods of stimulation using the annulus electrodes with and without a coating of TiN. If histology shows that larger charge densities per phase can be used without significant tissue damage or degradation of the electrode, then smaller electrodes can be used so that the resolution of the device can be improved.

### Limitations

This study measured the threshold to evoke a motor response (whisking) in anesthetized rats. Increased depth of anesthetic has been shown to increase the threshold currents required to evoke motor movements (Gioanni and Lamarche, 1985; Tandon et al., 2008). During the experiments we found that we only had a limited period of time where we could evoke motor responses before the animal became too deeply anesthetized, thus only a small number of electrodes could be compared in each animal, it would be preferred to be able to compare a large number of electrodes in the same animal to limit inter-animal effects. Although in this study there was no noticeable difference between the thresholds required to evoke whisking in different animals.

Additionally, threshold was determined by observations of whisker movements by three experienced observers. While this method is subjective, it allows for confirmation that the stimulation was of a level to activate the complete pathway from the activation of cortical neurons to the movement of the whiskers. Nevertheless, future work that combines this subjective method with a more objective method of measuring thresholds, such as measuring neuronal responses, may be used to more accurately and repeatedly compare the thresholds of electrodes with different GSAs.

## Conclusion

This study highlights the importance of choosing an appropriate electrode GSA and material in order to ensure that electrical stimulation will be both effective and safe. The results of this study indicate that to ensure the potential across an electrode is kept within the water window during effective stimulation, the GSA of an uncoated smooth PtIr electrode will need to be greater than 38,000 μm^2^, the largest GSA tested here. By comparison, if the electrode is coated with a thin film of TiN, the electrode’s surface area can be reduced to 16,000 μm^2^. Future work is needed that examines the histological damage observed with chronic stimulation using electrodes with these GSAs with and without a coating of TiN to ensure that GSAs determined here are safe to use chronically.

## Conflict of Interest Statement

Through Monash University, the researchers each may have a small share in the intellectual property of the project, which may be valuable if the project reaches a commercial stage.
