# Erratum: *In vivo* comparison of the charge densities required to evoke motor responses using novel annular penetrating microelectrodes

**DOI:** 10.3389/fnins.2015.00271

**Published:** 2015-07-29

**Authors:** Frontiers Production Office

**Affiliations:** Frontiers Production Office, FrontiersLausanne, Switzerland

**Keywords:** neural prosthesis, microelectrodes, charge injection capacity, *in vivo*, neural stimulation

Reason for Erratum:

Due to a production error the article was erroneously published in Frontiers in Neuroengineering, instead of Frontiers in Neuroscience. This mistake does not change the scientific conclusions of the article in any way and the publisher apologizes for the error.

Old citation:

*In vivo* comparison of the charge densities required to evoke motor responses using novel annular penetrating microelectrodes *by Brunton EK, Winther-Jensen B, Wang C, Yan EB, Hagh Gooie S, Lowery AJ, and Rajan R (2015). Front. Neuroeng. 8:5. doi: 10.3389/fneng.2015.00005*

The original article has been updated.

## References

[B1] Brunton E. K. Winther-Jensen B. Wang C. Yan E. B. Hagh Gooie S. Lowery A. J.. (2015). *In vivo* comparison of the charge densities required to evoke motor responses using novel annular penetrating microelectrodes. Front. Neurosci. 9:265. 10.3389/fnins.2015.00265 26283905 PMC4518750

